# Quantitative prediction of mixture toxicity of AgNO_3_ and ZnO nanoparticles on *Daphnia magna*

**DOI:** 10.1080/14686996.2020.1766343

**Published:** 2020-06-16

**Authors:** Min Jeong Baek, Jino Son, Jayoung Park, Yohan Seol, Baeckkyoung Sung, Young Jun Kim

**Affiliations:** a KIST Europe Forschungsgesellschaft mbH, Saarbrücken, Germany; b Ojeong Ecoresilience Institute, Korea University, Seoul, Republic of Korea; c Division of Energy & Environment Technology, University of Science & Technology, Daejeon, Republic of Korea

**Keywords:** Predictive nanotoxicology, mixture toxicity, zinc oxide nanomaterial, silver ion, concentration addition, independent action, Daphnia magna, 106 Metallic materials, 404 Materials informatics / Genomics, 503 TEM, STEM, STEM, 600 Others

## Abstract

Once metal-based engineered nanoparticles (NPs) are released into the aquatic environment, they are expected to interact with other existing co-contaminants. A knowledge gap exists as to how the interaction of NPs with other co-contaminants occurs. Here we selected ZnO NPs among various NPs, with Ag ion existing as a contaminant in the aquatic environment by Ag NPs widely used. A novel modeling strategy was demonstrated enabling quantitative and predictive evaluation of the aqueous mixture nanotoxicity. Individual and binary mixture toxicity tests of ZnO NPs and silver (as AgNO3) on Daphnia magna were conducted and compared to determine whether the presence of Ag ions affects the toxicity of ZnO NPs. Binary mixture toxicity was evaluated based on the concentration addition (CA) and independent action models. The CA dose-ratio dependent model was found to be the model of best fit for describing the pattern of mixture toxicity. The MIX I and MIX III suspensions (higher ratios of ZnO NPs to AgNO3) showed a synergism, whereas the MIX II suspension (lower ratio of ZnO NPs to AgNO3) showed an antagonism. The synergistic mixture toxicity at higher ratios of ZnO NPs to AgNO3 was caused by either the physiological or metabolic disturbance induced by the excessive ionic Zn or increased transport and accumulation in D. magna via the formation of complex of ionic Ag with ZnO NPs. Therefore, the toxicity level contributed via their aggregation and physicochemical properties and the dissolved ions played a crucial role in the mixture toxicities of the NPs.

## GRAPHICAL ABSTRACT

**Figure:**
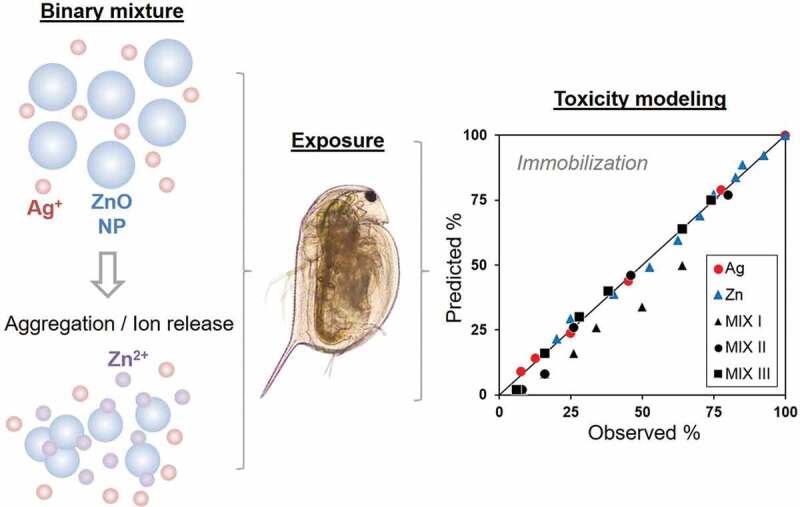

## 1. Introduction

Engineered inorganic nanoparticles (NPs) have been widely applied in a variety of fields because of their enhanced physicochemical and biological properties as well as their distinct functionalities compared to their counterpart bulk materials [1–7]. Given the widespread use of products containing metal or metal oxide NPs and the augmented number of applications, it is expected that the amount of metal-based engineered NPs released into the aquatic environment will increase.

Once the metal-based NPs are released into the aquatic environment, they undergo a different degree of dissolution and agglomeration depending on their characteristics and the ambient aqueous conditions [8], which will eventually affect their fate and toxicity [9]. The toxicity of metal-based NPs can be produced by their particulate properties, by dissolved ions released from NPs, or by both; therefore, it is challenging to explain how each of these fractions/forms contributes to the overall toxicity of the NPs. A recent study has shown that the relative contribution of released ions from NPs and the particulate fraction of copper (Cu) NPs and zine oxide (ZnO) NPs to the mortality of *Daphnia magna* varied greatly with the concentration of NPs [10]. These authors showed that the ions released from the NPs played an important role in toxicity and accumulation at low concentrations, whereas the particulate fraction played a crucial role in toxicity and accumulation at high concentrations.

Another critical issue related to the fate and toxicity of metal-based NPs is that they are rarely found individually, but rather as complex mixtures in the environment. Once metal-based NPs are released into the aquatic environment, they interact with other co-contaminants existing in the milieu, which, in turn, can lead to toxicity that differs from that of the individual chemicals. To date, many studies have reported on the adverse effects of individual NPs on aquatic organisms [11]; however, the toxicity of mixtures of NPs and co-contaminants has only recently been investigated (e.g. [12],). There are several contradictory studies that state that metal oxide NPs have synergistic [13,14] or antagonistic effects [15] on the bioavailability and toxicity of contaminants in daphnids. This inconsistency between studies is due to the differences in NPs, test species used, exposure conditions, and complex interactions that may occur between NPs and co-contaminants. However, these studies have mainly focused on how the presence of NPs at a specific concentration affects the toxicity of co-contaminants. There is still a lack of knowledge regarding the effect of mixture toxicity for the NPs and co-contaminants.

The two most widely used models for predicting mixture toxicity are the concentration addition (CA) and independent action (IA) models, which assume the same mode of action (MoA) and different MoA, respectively [16]. While these two models are the most commonly used reference models for describing and predicting mixture toxicity, there are several other models that consider synergistic or antagonistic interactions that may occur between chemicals, or even more sophisticated models such as the dose-level or dose-ratio dependent responses of two chemicals in a mixture [16]. However, in many cases, accurate information about the MoA of the individual chemicals in a mixture is limited, which is true for most metal-based NPs. In general, the CA model is frequently applied as a general ‘rule of thumb’ for predicting the toxicity of a mixture because of its conservative estimate relative to the IA model; however, it may be desirable to select a model that best describes and predicts the toxicity of the mixture when the MoA of the individual chemical is not well known.

Among the various metal-based NPs, ZnO and Ag NPs have gained great attention over the last years [17]. ZnO NPs have been applied to various products including medical devices, pharmaceutical agents, drug carriers, optoelectronics, cosmetics, catalysts, ceramics, and pigments [18]. Silver (Ag) is a naturally occurring metallic material that is one of the most toxic metals found in the freshwater environment [19,20]. The regulatory authorities have been actively regulating Ag and the predicted environmental concentration ranges from 0.03 μg/L to 1 μg/L [21,22]. In addition, the widespread applications of Ag NPs in recent years have accelerated Ag contamination in aquatic environments, especially through facilitated ionization in the water-rich milieu. Therefore, as the general situation involves ZnO and Ag NPs being present as a mixture in marine and freshwater environments, an improved understanding of how the interaction between ZnO NPs and Ag ions affects aquatic organisms is essential for both regulatory purposes in environmental risk assessment and the application of safe-by-design methodology. Lopes et al. [17] investigated the binary mixture of two forms of the same element with different forms (NP and ion) such as the ZnO NPs-Ag NPs, ZnO NPs-ZnCl_2_, Ag NPs-AgNO_3_, ZnCl_2_-AgNO_3_, and demonstrated that the Ag ion was responsible for a synergistic pattern in the Ag NP-AgNO_3_ mixture.

In the present study, *D. magna* was selected as a model organism, which has been commonly used as a representative aqueous species for assessing the toxic effects of chemicals and nanoparticles. The current European regulatory guidelines also recommend the evaluation of chronic toxic effects of active ingredients in pharmaceutics by using the model of *D. magna* [23]. The effects of ZnO NPs and Ag ion (as AgNO_3_), alone or in combination, on the immobilization of *D. magna* were determined after 48 h of exposure in ISO test water (= ISO medium). The concentration of individual chemicals [expressed as a toxic unit (TU), where 1 TU = EC_50_] in the mixture was determined using the effective concentration (EC_50_ value) of each chemical causing 50% immobilization to *D. magna* obtained from the individual toxicity test. The sum of TU (TU_mix_, a dimensionless ratio) of each chemical in the mixture ranged from 0.06 TU_mix_ to 2.0 TU_mix_, and each chemical in the mixture was adjusted to three different TU ratios (7:3, 5:5, and 3:7). The observed mixture toxicity effects were compared with the predicted toxicity effects based on both the CA and IA reference models using the MIXTOX model of Jonker et al. [16].

## 2. Materials and methods

### 2.1. Test chemicals

AgNO_3_ (purity 99.99%) and ZnO nanopowders (˂ 50 nm size, purity ≥ 99%) were purchased from Alfa Aesar (Alfa Aesar GmbH and CoKG, Karlsruhe, Germany). The AgNO_3_ was dissolved in Milli-Q water and the ZnO nanopowder was suspended in Milli-Q water. The ISO medium was composed of 294 mg/L CaCl_2_ · 2H_2_O, 123.25 mg/L MgSO_4_ · 7H_2_O, 64.75 mg/L NaHCO_3_, 5.75 mg/L KCl, and 2 µg/L Na_2_SeO_3_ (OECD, 2004). A stock suspension of ZnO NPs was freshly prepared in the medium by taking the exact volume from suspension while stirring and was then subjected to sonication for 30 min in an ultrasonic bath at 45 kHz with 240 W (VWR USC1700TH, Germany). Three binary stock mixtures of AgNO_3_ and ZnO NPs were prepared at a mixture toxicity strength of 2.0 (i.e. sum of toxicity of toxic unit (∑TU) = 2.0, where TU is defined as the fraction of the individual compound to its EC_50_ value obtained in an individual toxicity test), while the TU ratio of AgNO_3_ to ZnO NPs in the mixture was maintained at 5:5, 3:7, or 7:3 (hereafter referred to as MIX I, MIX II, or MIX III, respectively). Additionally, the ZnO NPs suspensions (ZnO NPs I, II, and III) applied individually with the same amount of ZnO NPs as in the three binary stock mixtures were prepared. All stock solutions and suspensions were maintained in an incubator at a temperature of 20.0 ± 0.5°C for 24 h and were then subjected to sonication for 5 min before being used for subsequent dilution. For inductively coupled plasma mass spectroscopy (ICP-MS) analysis, ion calibration standards were prepared using the Ag standard solution (Spex Certiprep Standards, Metuchen, New Jersey, USA) and nitric acid (65% or 70%, Sigma-Aldrich, St. Louis, Missouri, USA) was used for sample digestion.

### 2.2. Test organism

*D. magna* ephippia were obtained from Daphtoxkit F™ (MicroBioTests Inc., Gent, Belgium). Once hatched, the neonates were transferred to a 2 L glass beaker containing 1.4 L ISO medium. All glass beakers were kept in an incubator at a temperature of 20.0 ± 0.5°C with a 16:8 h light:dark exposure. The daphnids were fed with *Chlorella vulgaris* at a concentration of 3.0 × 10^5^ cells/mL with a supplement of YCT (a mixture of yeast, cerophyll, and trout chow fish food) twice a week. Neonates (< 15 h old) obtained from the fifth brood were used in the toxicity tests to minimize variability.

### 2.3. Characterization of ZnO NPs in ISO medium

The hydrodynamic diameter (HDD), zeta potential, and free Zn^2+^ ion from ZnO NPs over the different incubation times (0, 24, and 48 h) were investigated for each individual ZnO NP and binary stock mixture (AgNO_3_ + ZnO NP) suspensions. After 24 h of sample preparation, all suspensions were sonicated again for 5 min (indicated as time zero) and 1 mL was taken from each suspension as the zero-time sample for characterization. A total of 10 mL of each suspension was dispensed into 6-well plates (Cellstar®, Greiner Bio-one, Germany). After 24 and 48 h of incubation, 1 mL of each sample was taken from the mid-height of the 6-well plates and used for the characterization. All measurements were conducted in triplicate.

The HDD and zeta potential of ZnO NPs in each suspension were measured using a dynamic light scattering analyzer (ZetaSizer Nano ZS, Malvern Instruments Ltd., UK). For quantification of the Zn ion in each suspension at each incubation time, 500 μL of the sample was taken from each suspension and filtered by centrifugation at 13,300 × *g* using a 0.5 mL Amicon filtration unit (MWCO 3 kDa, Amicon Ultra-0.5). The supernatants were acidified with nitric acid and subjected to metal analysis using an ICP-MS (iCAP Q ICP-MS, Thermo Fisher Scientific, Germany).

The shape and size of the ZnO NPs and ZnO/AgNO_3_ in the medium were characterized using a scanning electron microscope (Quanta FEI-SEM, Holland). A total of 20 μL of each sample was deposited on a silicon wafer for SEM analysis. Afterward, the silicon wafer was washed with distilled water for desalting and fixation. The ZnO NP samples collected at 0, 24 and 48 h were deposited onto a silicon wafer and then dried for 3 h at room temperature.

### 2.4. Analysis of Ag and Zn accumulations in *D. magna*

The *D. magna* was exposed to the control and mixture solutions for 6 hours under the standard acute toxicity conditions. Considering the sensitivity limit of the ICP-MS, all concentrations were increased to 5 times. Five *D. magna* were placed in each 100 mL glass beaker containing 20 mL of test solution. After 6 hours, *D. magna* were removed from each of the sample medium, rinsed with 100 mL of deionized water for about 2 min, then transferred to a glass vial. The *D. magna* were subsequently dried at 60°C and digested in 4 mL of HNO_3_ (69%, Sigma-Aldrich, St. Louis, Missouri, USA) and in 1 mL of H_2_O_2_ (30%, Sigma-Aldrich, St. Louis, Missouri, USA) at 100°C. Four hours later, after cooling down and dilution of the samples to 5% HNO_3_, the Zn and Ag concentrations were measured by an ICP-MS.

### 2.5. Single toxicity test

An acute immobilization test with *D. magna* was conducted according to the OECD guideline 202 (OECD, 2004). The nominal test concentrations of AgNO_3_ and ZnO NPs ranged from 0.5 μg AgNO_3_/L to 2.5 μg AgNO_3_/L and 0.04 mg ZnO/L to 20 mg ZnO/L, respectively. For each test concentration and control, eight replicates with five neonates each (< 15 h old) were performed in 6-well plates (Cellstar®, Greiner Bio-one), containing 10 mL of either test solution or suspension. The plates were covered and incubated for 48 h at 20 ± 1°C in the dark. After 48 h, the numbers of immobilized daphnids were analyzed.

### 2.6. Binary mixture toxicity test

Binary mixture toxicity tests were performed using a procedure similar to that used in the individual toxicity test. A total of seven different ∑TU levels ranging from 0.0625 to 2.0 were prepared by serial dilution of each stock mixture with the medium, while the TU ratios of AgNO_3_ to ZnO NPs at each ∑TU level were maintained at 5:5 (MIX I), 3:7 (MIX II), or 7:3 (MIX III). After 48 h exposure, the numbers of immobilized daphnids were analyzed.

### 2.7. Data analysis

For data obtained from individual exposures, 48 h EC_50_ values for immobilization with 95% confidence intervals were calculated using the drc package of R [24].

Data obtained from the binary mixture toxicity tests were analyzed by comparing the observed data with the predicted mixture effects from the two reference models: CA, which assumes concentration-additive cumulative toxicity and IA, which assumes response-additive cumulative toxicity, using the MIXTOX tool proposed by Jonker et al. [16]. The conceptual reference models, CA and IA, were used in Equations (1) and (2), respectively.

(1) $$\overset{n}{\underset{i=1}{\sum }}\left(\frac{C_{i}}{EC_{x_{i}}}\right)=1$$

where, *C_i_* is the concentrations of the chemical *i* in the mixture and *EC_xi_* is the effective concentration of the chemical *i* causing 50% effect. This quotient expressed as the TU (TU = C_i_/ECx_i_) represents the concentration of mixture components as fractions of equivalent effective concentrations of the individual chemical.

(2) $$y=u_{max}\overset{n}{\underset{i=1}{\prod }}q_{i}\left(C_{i}\right)$$

where, *y* is the response variable, *q_i_*(*C_i_*) is the probability of the non-response at concentration *c* of chemical *i, u_max_* is the response in the control group, and ∏ is the multiplication function. Both reference models, CA and IA, were extended by stepwise addition of extra parameters into the deviation functions to describe synergistic/antagonistic interactions (S/A), and dose-ratio (DR) or dose-level (DL) dependent deviation [16]. In the S/A model, the parameter ‘*a*’ can be negative or positive for both reference models. When *a* = 0, the S/A model can be reduced to the CA or IA models. For a DR dependent deviation, an additional parameter ‘*b*_DR_’ was included in the deviation function to evaluate whether the deviation from either reference model was dependent on the composition of the mixture. A parameter ‘*b*_DR_’ quantifies the degree of decreased (antagonism if *b*_DR_ > 0) or increased (synergism if *b*_DR_ < 0) toxicity due to the lead chemical, and parameter ‘*a*’ quantifies the degree of increased (antagonism if a > 0) or decreased (synergism if a < 0) toxicity in the rest of the mixture. If ‘*a*’ and ‘*b*_DR_’ have opposite signs, a switch between antagonism and synergism occurs; however, if they have the same sign, the magnitude of the antagonism or synergism varies with the ratio of chemicals, but a switch between antagonism and synergism does not occur [16,25]. For a DL dependent deviation, the additional parameter ‘*b_DL_*’ was included together with the parameter ‘*a*’ into the deviation function. DL describes antagonism or synergism depending on the doses of each chemical in the mixture. The value of ‘*a*’ indicates the deviation at low doses and the value of ‘*b*_DL_’ indicates at what dose level the deviation changes (i.e. from synergism to antagonism or vice versa). When *a* < 0, synergism occurs at a low dose level and antagonism occurs at a high dose level, whereas the opposite occurs if *a* > 0. For CA-DL, the deviation change occurs at a lower dose level than EC_50_ (if *b*_DL_ > 1), EC_50_ level (if *b*_DL_ = 1), or higher dose level than EC_50_ (if 0 < *b*_DL_ < 1). If *b*_DL_ = 0, the equation reduces to the S/A model and if *b*_DL_ < 0, the magnitude of synergism/antagonism (‘*a*’) becomes DL, but does not switch. For IA-DL, the deviation change occurs at a lower dose level than EC_50_ (if *b*_DL_ > 2), EC_50_ level (if *b*_DL_ = 2), and higher dose level than EC_50_ (if 1 < *b*_DL_ < 2). If *b*_DL_ = 0, the deviation function again reduces back to the S/A model and if *b*_DL_ < 1, the magnitude of synergism/antagonism becomes response‐dependent but does not switch [16,25]. These extended models were fitted to the data using the method of maximum likelihood while minimizing the sum of the squared residuals by running the Solver Function in Microsoft® Excel. The statistically significant improvement in the model fit of the extended models was analyzed using chi-squared (χ^2^) testing. For details on the derivation of these deviation functions, refer to Jonker et al. [16].

Statistical differences in HDD, zeta potential, and released Zn^2+^ among treatments (I, II, and III) for each individual and mixture suspension at each incubation time (0, 24, and 48 h) were analyzed by one-way ANOVA, followed by a Tukey’s HSD test for multiple comparisons. The same statistical analysis was performed to compare the difference in these variables between incubation times within each individual and mixture suspension treatment and to compare the differences between the individual and mixture administered to the same Zn level at each incubation time (SAS version 9.4).

## 3. Results and discussion

### 3.1. Physicochemical characterization of ZnO NPs in individual and mixture suspensions

The HDD, zeta potential, and released Zn ion in each of the three types of individual and mixture suspensions among the different incubation times (0, 24, and 48 h) are shown in Table 1.

**Table 1.** Hydrodynamic diameter (HDD), zeta potential, and released zinc ion (relative amount (%) of dissolved Zn ion) in each of the three types of individual (ZnO NPs alone; ZnO I, II, and III) and mixture (AgNO_3_+ ZnO NPs; MIX I, II, and III) suspensions over incubation time (0, 24, and 48 h). Each mixture suspension was prepared at a toxic unit (∑TU) = 2.0, while the TU ratio of AgNO_3_ to ZnO NPs in the mixture was kept at 5:5 (MIX I), 3:7 (MIX II), or 7:3 (MIX III). ZnO NPs suspensions (ZnO NPs I, II, and III) applied singly with the same amount of ZnO NPs as in the three binary stock mixtures were prepared. See also Figure S1 in Supplementary information.

	Hydrodynamic diameter (nm)			Zeta potential (mV)			Released zinc ion (μg/ml Zn) (relative amount (%) of dissolved Zn ion)		
	0 h	24 h	48 h	0 h	24 h	48 h	0 h	24 h	48 h
**ZnO I**	974 ± 257 a	777 ± 151 a	940 ± 74 a	−3.0 ± 1.6 a	−11.8 ± 2.2 b	−8.2 ± 1.2 b	0.56 ± 0.03 c (24.6 ± 1.5)	0.96 ± 0.01 b (42.1 ± 0.7)	1.07 ± 0.02 a (46.6 ± 0.9)
**MIX 1**	547 ± 116 a	679 ± 273 a	718 ± 322 a	−0.6 ± 1.6 a	−13.6 ± 0.8 b	−12.9 ± 2.1 b	0.53 ± 0.01 b (23.2 ± 0.5)	1.03 ± 0.03 a (45.0 ± 1.4)	1.10 ± 0.04 a (48.0 ± 1.7)
**ZnO II**	945 ± 328 a	789 ± 253 a	817 ± 301 a	−5.5 ± 0.6 a	−8.3 ± 1.5 ab	−9.4 ± 1.4 b	0.60 ± 0.02 b (43.9 ± 1.2)	0.73 ± 0.02 a (53.0 ± 1.4)	0.72 ± 0.03 a (52.7 ± 2.1)
**MIX II**	827 ± 132 a	717 ± 210 a	482 ± 83 a	−4.5 ± 1.5 a	−10.1 ± 0.9 b	−12.2 ± 1.2 b	0.65 ± 0.08 b (47.8 ± 5.7)	0.79 ± 0.01 a (57.6 ± 0.7)	0.70 ± 0.03 ab (51.4 ± 2.2)
**ZnO III**	730 ± 90 a	1149 ± 129 b	817 ± 133 a	1.7 ± 0.4 a	−12.3 ± 1.8 c	−9.0 ± 1.1 b	0.70 ± 0.02 c (21.9 ± 0.6)	1.09 ± 0.01 b (34.1 ± 0.5)	1.23 ± 0.02 a (38.4 ± 0.7)
**MIX III**	642 ± 183 a	950 ± 139 a	729 ± 172 a	1.9 ± 0.7 a	−14.4 ± 1.5 c	−10.2 ± 1.7 b	0.67 ± 0.02 c (21.0 ± 0.6)	1.10 ± 0.06 b (34.1 ± 1.9)	1.24 ± 0.02 a (38.5 ± 0.5)

* Different letters indicate a statistical difference in HDD, zeta potential, and released Zn^2+^ between incubation times within the treatment of each individual and mixture suspension after one-way ANOVA, followed by a Tukey’s HSD test for multiple comparisons.

The HDDs of ZnO NPs in the individual treatments were in the range of 730.3–1149 nm, while those in the mixture were 482–950 nm. No significant change in the HDD with incubation time was observed in either individual or mixture suspensions, except for the ZnO III suspension. Therefore, ZnO NPs were rapidly agglomerated in the medium, even when measured immediately after sonication, and the HDD of the ZnO NPs was constant in the medium for the duration of the experiment, regardless of individual or mixture suspensions. When comparing the HDD in the individual suspensions to that in the corresponding mixture suspensions at the same incubation time, no statistically significant difference was found for any of the incubation times; thus, the presence of the ionic Ag in the mixture did not affect the HDD of the ZnO NPs. Compared with previous studies that measured a similar size of ZnO NPs in the medium, the HDD of the ZnO NPs measured in the present study after 0 h of incubation was similar to that of the 1061 nm reported by Lopes et al. [26]; however, the HDD of the ZnO NPs measured after 48 h of incubation was smaller than the 4533 nm that was reported by these authors. Possible explanations for this difference include the differing stock preparation methods (such as sonication and stock concentration) and the source of the NPs (probably related to different methods for synthesizing NPs).

**Figure 1.**
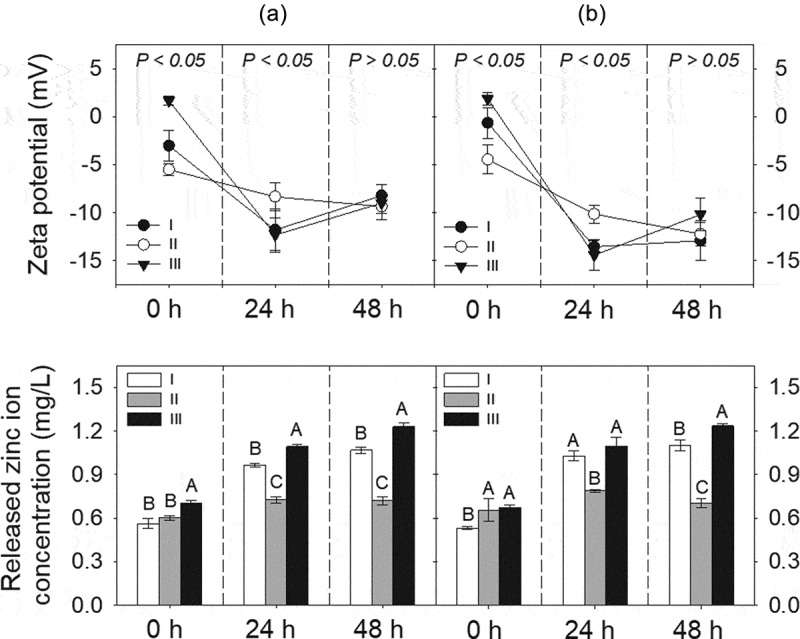
Time-dependent changes in zeta potential and released zinc ion concentration in individual (ZnO NPs alone; a) or mixture (AgNO_3_ + ZnO NPs; b) suspensions. Each mixture suspension was prepared at a toxic unit (∑TU) = 2.0, while the TU ratio of AgNO_3_ to ZnO NPs in the mixture was kept at 5:5 (MIX I), 3:7 (MIX II), or 7:3 (MIX III). ZnO NPs suspensions (ZnO NPs I, II, and III) applied singly with the same amount of ZnO NPs as in the three binary stock mixtures were prepared. The *P*-value in the top row graph indicates statistical differences in the zeta potential value among treatments at each incubation time. Different letters in the bottom row graph indicate significant differences in the released zinc ion among treatments at each incubation time.

The zeta potential of the ZnO NPs in individual and mixture suspensions ranged from −12.3 to 1.7 mV and from −14.4 to 1.9 mV, respectively. The zeta potential values decreased with increasing incubation time (*P* < 0.05), regardless of individual and mixture suspensions. Considering that the absolute values for the zeta potential of the ZnO NPs were less than 30, the ZnO NPs were gradually dispersed but not well dispersed in the medium. Additionally, there was a significant difference in the zeta potential values measured at 0 and 24 h among the treatments in both the individual and mixture suspensions, but not at 48 h (Figure 1). When comparing the zeta potential value in the individual suspensions to that in the corresponding mixture suspensions at the same incubation time, no statistically significant difference was found, except for between ZnO NPs I and Mix I at 48 h.

**Figure 2.**
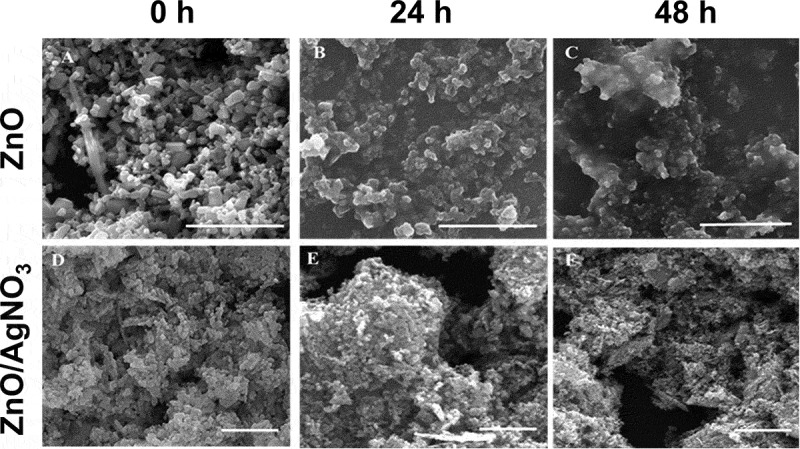
SEM images of ZnO NPs (a, b, and c) and ZnO/AgNO_3_ (d, e, and f) suspensions prepared at a concentration of Mix III in ISO medium at 0, 24 and 48 h of incubation, respectively. Scale bar = 2 µm.

**Figure 3.**
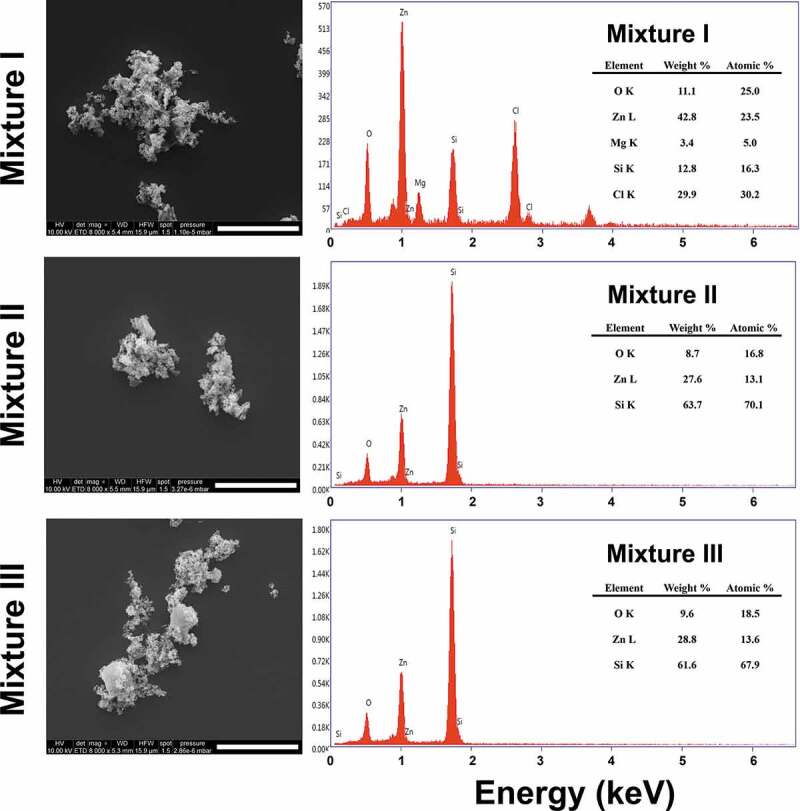
Representative SEM images and EDX results of 100-fold mixture I, II, and III. Scale bar = 5 µm.

The released Zn ion from the ZnO NPs at 0 h incubation time in the individual and mixture suspensions ranged from 0.56 (ZnO NPs I) to 0.70 mg/L (ZnO NPs III), and from 0.53 (MIX III) to 0.67 mg/L (MIX II), respectively. However, when comparing the percentage of Zn ion released relative to the initial concentration at the 0 h incubation time, the higher the initial concentration of ZnO NPs, the greater the amount of Zn ion released, regardless of individual and mixture suspensions. This was mainly due to the difference in the initial concentration of ZnO NPs among the treatments. The amount of Zn ion released from the ZnO NPs increased with increasing incubation time (*P* < 0.05). When comparing the treatments at the same incubation time in individual or mixture suspensions (Figure 1), there were significant differences not only in the released Zn ion from ZnO NPs but also in the percentages of Zn ion released relative to the initial concentration of ZnO NPs. However, the amount of Zn ion released did not differ between the individual and mixture suspensions with the same initial concentration of ZnO NPs, except for ZnO NPs I/MIX I and ZnO NPs II/MIX II at 24 h. Xiao et al. [10] reported that the concentration of NPs is a crucial factor affecting the dissolution characteristics of NPs. The percentage of Zn ion released from ZnO NPs applied in the present study was comparable to Li et al. [14] who reported that 30% at 0.5 mg/L of ZnO NPs and 20% at 2 mg/L of ZnO NPs were released after 24 h of incubation in a simplified M7 medium. Xiao et al. [10] reported that 59% of the ZnO NPs at 1 mg/L was released after 1 h of incubation in ISO medium. However, the Ag ion concentrations in the mixture suspensions (I, II, and III) could not be measured because of their low value that was below the detection limit. Thus, to investigate whether the presence of AgNO_3_ affects Zn ion release from ZnO NPs, the concentrations of each component in the mixture suspension was increased 100-fold. Additionally, three individual suspensions with 100-fold ZnO NPs were prepared. Each of the 100-fold individual or mixture suspension was prepared as described in section 2.1. The results showed that a relatively low amount of Zn ion (approximately 0.8%−2% of dissolution relative to the initial concentration) was released from the suspensions of 100-fold ZnO NPs. A higher amount of Zn ion was released in all 100-fold mixture suspensions compared to the 100-fold ZnO NPs suspensions. Additionally, the amount of Ag ion in the mixture suspensions was lower than that applied in the mixture suspension. Therefore, ionic Ag can be adsorbed onto the surface of ZnO NPs, which, in turn, may affect the equilibrium of dissolved and adsorbed Zn ions as well as the competition of ionic Zn and Ag in the medium. Figure 2 shows the SEM images of ZnO NPs III and MIX III at different incubation times. ZnO NPs in both individual and mixture suspensions exhibited an irregular shape and large agglomerates as the incubation time increased. Nevertheless, unlike the ICP results described above, energy-dispersive X-ray (EDX) analysis of the all mixture samples did not confirm the presence of Ag ions on the surface of the ZnO NPs (Figure 3).

### 3.2. *Single toxicity of AgNO_3_ and ZnO to the immobilization of* Daphnia magna

The 48 h EC_50_ values for AgNO_3_ and ZnO NPs were 0.89 (0.76–1.03) μg/L and 2.28 (1.93–2.74) mg/L, respectively. This indicates that ionic Ag had a higher toxicity effect on the immobilization of *D. magna*, being more than three orders of magnitude more toxic than that of ZnO NPs. The EC_50_ value of Ag reported here was comparable to that of 1.05 μg/L in ASTM medium reported by Ribeiro et al. [27]. The EC_50_ value of ZnO NPs obtained in the present study was also comparable to the finding of Heinlaan et al. [28] who reported an EC_50_ value of 3.2 mg/L for ZnO NPs (with a nominal size of 50–70 nm) in ISO medium, but was slightly higher than those of Lopes et al. [26] who reported an EC_50_ value of 1.02 mg/L for ZnO NPs (with a nominal size of 30 nm) and 1.10 mg/L for ZnO NPs (with a nominal size of 80–100 nm) in ASTM medium.

Several studies have reported that the factor contributing to the toxicity of metal oxide and metal-based NPs is mainly attributed to their dissolution properties [8,29]. However, it is not well understood how much the dissolved Zn ion contributes to the toxicity of the ZnO NPs and what the underlying mechanisms are. The degree of ion dissolution from metal-based NPs differs depending not only on the characteristics of the NPs themselves but also on the various surrounding environmental parameters of the exposure media (such as pH, temperature, and organic matter). Given the relatively low toxicity of ZnO NPs compared with their bulk counterparts and Zn salts reported in the literature [30], the toxicity of ZnO NPs cannot be explained solely by the released Zn ions from the ZnO NPs present in the exposure media and can be at least somewhat explained, if not fully, by the particulate form.

### 3.3. *Mixture toxicity of AgNO_3_ and ZnO NPs for the immobilization of* Daphnia magna

In the present study, two reference models (CA and IA) were tested to assess the immobilization response of *D. magna* when exposed to mixtures of AgNO_3_ and ZnO NPs. All parameters and statistics obtained by fitting the MIXTOX tool are shown in Table 2.

**Table 2.** Summary of the model fits for the mixture toxic effect of AgNO_3_ and ZnO NPs on immobilization of *Daphnia magna*. SA is synergism/antagonism, DL is dose level-dependent deviation from the reference, and DR is dose ratio-dependent. C_max_ is the control response; β is the slope of the individual dose-response curve; EC_50_ is the median inhibition concentration; *a* and *b* are parameters in the deviation functions. SS (sum of squared residuals) is the objective function; χ ^2^ coefficient of determination factor and *p* (χ ^2^) indicates the outcome of the likelihood ratio test. NA means that the quantity is not applicable.

	Concentration addition				Independent action			
Parameter	CA	S/A	DR	DL	IA	S/A	DR	DL
C_max_	99.12	98.95	98.05	99.74	98.97	98.32	98.20	98.53
β_AgNO3_	2.38	2.38	2.37	2.30	2.24	2.31	2.17	2.30
β_ZnO NPs_	0.68	0.68	0.69	0.64	0.65	0.68	0.66	0.68
EC_50 AgNO3_	0.91	0.92	0.91	0.91	0.94	0.96	0.92	0.95
EC_50 ZnO NPs_	2.21	2.29	2.60	2.13	2.16	2.32	2.46	2.30
*SS*	534.7	532.1	437.2	509.8	664.5	658.9	515.2	656.7
a	NA	−0.13	−2.39	0.65	NA	−0.20	−1.57	−0.35
b_DR_	NA	NA	3.74	NA	NA	NA	2.98	NA
b_DL_	NA	NA	NA	0.83	NA	NA	NA	1.03
R^2^	0.98	0.98	0.98	0.98	0.97	0.97	0.98	0.97
*p*(χ^2^)	NA	0.68	0.03	0.43	NA	0.59	0.01	0.81

**Figure 4.**
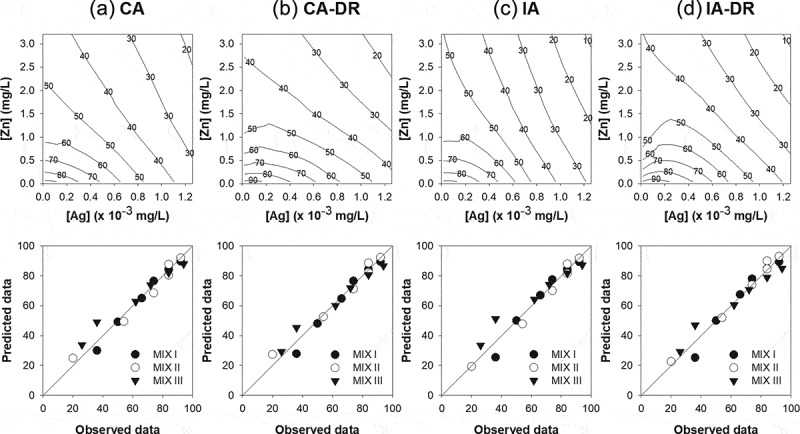
Reference model and the model of best fit (dose-ratio dependent deviation) for both concentration addition (first two columns; a and b) and independent action (last two columns; c and d) describing the mixture toxic effect of AgNO_3_ (× 10^−3^ mg/L) and ZnO NPs (mg/L) on the immobilization of *Daphnia magna*. The value on the isobolic contour line represents the mobilization rate predicted by each model. The bottom row graph represents the relationship between the predicted and observed mobilization rates in the three mixture suspensions with different TU ratios of AgNO_3_ to ZnO NPs (5:5, MIX I; 3:7, MIX II; and 7:3, MIX III). The straight line represents a 1:1 relationship between the predicted and observed mobilization rates.

The fitting of the mixture data to the CA model yielded a sum of squared residuals (*SS*) of 534.7 (Table 2 and Figure 4(a)). Extension of the CA model with the parameter ‘*a*’ into the CA-S/A model decreased the *SS* to 532.1, but this was not significant (Table 2), indicating no notable synergisms/antagonism. An additional extension of the CA-S/A model with parameter ‘*b*_DL_’ into the CA-DL reduced the *SS* but did not improve the model fit. Nevertheless, further extending the CA-S/A model with ‘*b*_DR_’ significantly reduced the *SS* compared to the other models, implying a DR dependent deviation. Specifically, the negative value of parameter *a* = −2.39 indicates that synergism was mainly caused when ZnO NPs were at higher concentrations in the mixture. The positive value of parameter *b*_DR_ = 3.74 indicates antagonism when ionic Ag increased in concentration and became dominant in the mixture, thus the shift between antagonism and synergism occurred when TU50_ZnO NPs_ = 0.57·TU50_AgNO3_, which is equivalent to [*C*_ZnO NPs_] = 1635·[*C*_AgNO3_]. Thus, the model of best fit was CA-DR, with the ionic Ag and ZnO NPs mixture demonstrating a DR dependent mixture toxicity (Figure 4(b)).

Similar results were obtained when the mixture data was applied to the IA model. This yielded an *SS* of 664.5 (Table 2 and Figure 4(c)). The IA model was further extended with parameter *a* to describe synergism/antagonism and parameter *a* and *b*_DL_ to describe DL dependent deviation, but neither parameters significantly improved the model fit (*p*[χ^2^] = 0.97 for IA-S/A, *p*[χ^2^] = 0.81 for IA-DL). However, as with CA, further extension of the IA-S/A model with *b*_DR_ significantly reduced the *SS* to 515.2, indicating a DR dependent deviation from IA. The negative value of *a* = −1.57 indicates synergism with a shift to synergism if the toxicity is dominated by ZnO NPs (*b_DR_* = 2.98). The shift between antagonism and synergism occurred when the ratio [*C*_ZnO NPs_]/[*C*_AgNO3_] exceeded 2204. Therefore, the model of best fit was achieved with IA-DR (Figure 4(d)), and thus the mixture of ionic Ag and ZnO NPs has DR dependent mixture toxicity.

**Figure 5.**
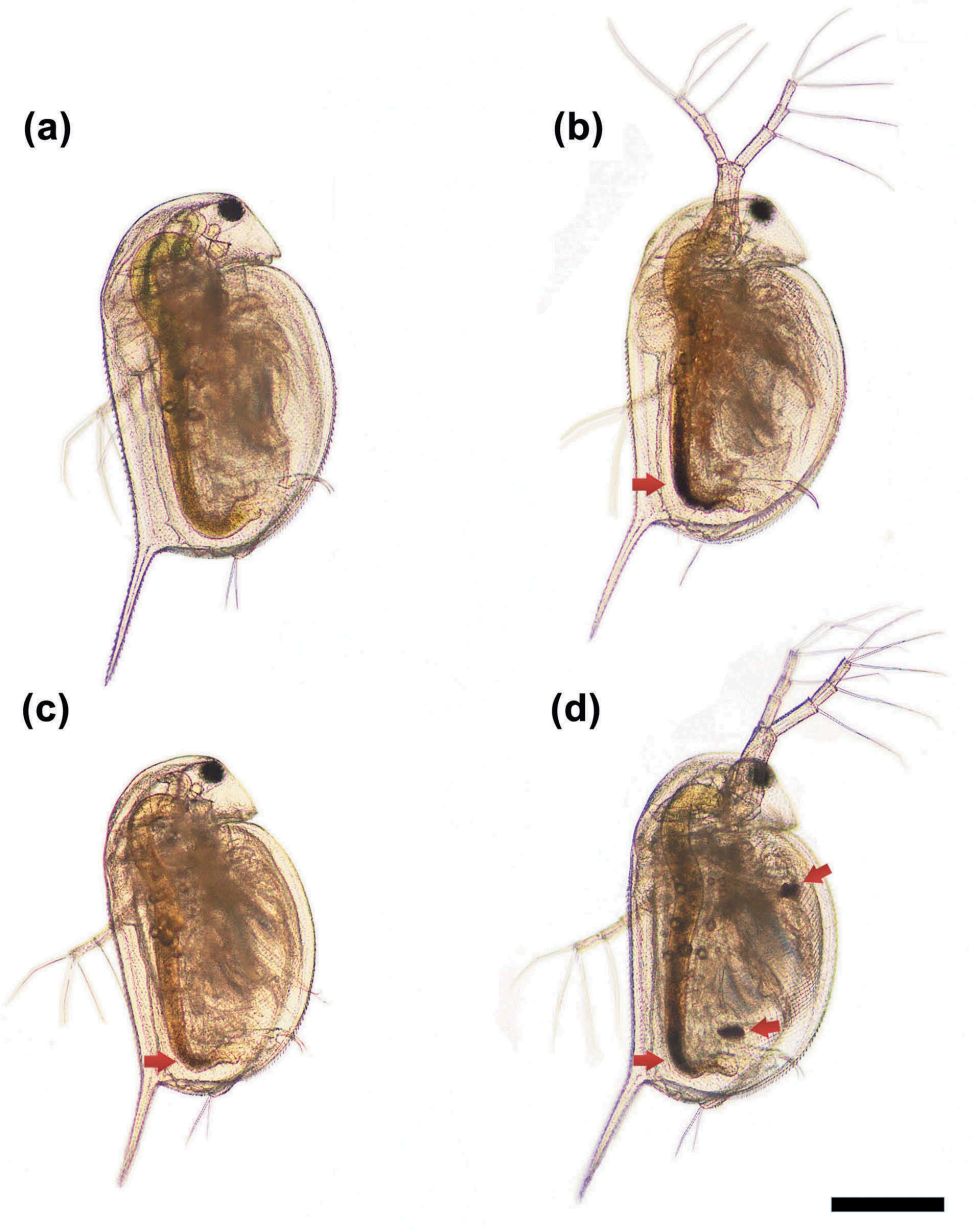
Representative bright-field microscopy images of *Daphnia magna* exposed to control (a) and 0.5 TU level with different TU ratios of AgNO_3_ to ZnO NPs (5:5, MIX I (b); 3:7, MIX II (c); and 7:3, MIX III (d)) for 48 h. The red arrows indicate the region in the gut where the nanoparticles are accumulated. Scale bar = 400 µm.

Together with the ICP-MS analysis data on the accumulated Zn and Ag ions in *D. magna* (Fig. S2), the representative light microscopy images of the organism exposed to a 0.5 TU level of each mixture suspension showed that a considerable number of NPs was taken up by *D. magna* (Figure 5). In particular, as evidenced by the dark regions of the NPs in the gut, more NPs were accumulated in *D. magna* when exposed to MIX I (Figure 5(b) and MIX III (Figure 5(d)) than MIX II (Figure 5(c)). This finding may partly explain the synergism for MIX I and III and antagonism for MIX II. The mixture toxicity of the ionic Ag and ZnO NPs to *D. magna* follows a DR dependent deviation pattern from the reference models, indicating a shift between synergism and antagonism that was dependent on the TU ratio of the mixture constituents. When modeled for the three mixture suspensions based on the DR dependent deviation function, MIX I and MIX III suspensions (low Ag ions) showed a synergism, while the MIX II suspension (high Ag ions) showed an antagonism. However, in the previous study, a synergistic effect was depicted when the concentrations of Ag ions increased in the mixture of Ag ions and Ag NPs, while antagonism was observed with Ag NPs increase in suspension [17].

In general, if the Zn ion is released from the ZnO NPs into the test media, it is expected that the Zn ion will interact or compete with not only ionic Ag but also other cations present in the medium, such as Ca^2+^ and Mg^2+^, for the binding sites. Such competition would occur regardless of the mixture suspensions applied in the present study. However, even though this is true, it is expected that these competitions would differ between suspensions. Considering the lower TU ratio of ZnO NPs to AgNO_3_ in MIX II than those in the other mixture suspensions, the competition between ionic Zn and dissolved ionic Ag would be more likely to occur in MIX II compared to the other mixture suspensions. Thus, it is postulated that the increased competition between ionic Ag, Zn, and other cations may cause an antagonistic effect in this mixture suspension.

The synergistic mixture toxicity at higher ratios of ZnO NPs to AgNO_3_ might be due to either physiological or metabolic disturbance induced by the excessive Zn ion or the increased transport and accumulation in *D. magna* via formation of ionic Ag-ZnO NPs complexes (a phenomenon that is termed the ‘Trojan horse effect’ [31,32]), or both. In the present study, the higher the concentration of ZnO NPs, the more Zn ion was released. As discussed above, this may increase the competition between Ag and Zn ions, but a greater amount of Zn ions than that required for physiological and metabolic processes can exert toxic effects. Such mixture toxicity effects may be synergistic rather than offsetting the toxicity at certain concentration ratios that overwhelm the competition between ions. This may be one reason for the synergism we found in the present study when the TU ratio of ZnO NPs to AgNO_3_ was increased in the MIX I and MIX III mixture suspensions. Another possible reason for the synergism that we found in the MIX I and MIX III may be the following. As discussed for the 100-fold mixture suspensions, the SEM-EDX analysis did not confirm the presence of Ag ions on the surface of ZnO NPs (Figure 3); however, the ICP results showed that ionic Ag could be adsorbed on the surface of ZnO NP and filtered. As the concentration of ZnO NPs increases, the ionic Ag in the test media may become more likely to adsorb on the surface of ZnO NPs. The hydroxyl group on the ZnO NPs surface may be a favorable site for the adsorption of Ag ions, which can play a role as a carrier of the Ag ion into the organism, i.e. Trojan horse-like effects [32]. This may lead to facilitated transport and accumulation of Ag-ZnO NPs complexes into *D. magna* and result in increased toxicity. Although this mechanism cannot be fully supported in the present study due to the failure of the confirmation of the presence of Ag ions on the surface of ZnO NPs, the possible uptake by *D. magna* as the complex of ionic Ag and ZnO NPs cannot be negligible as evident from the ICP results. However, we found information on the body of *D. magna* about the aggregation by the arrow in the Figure 5(d). ZnO NP demonstrated to sediment as micron sized ZnO aggregates in the medium. The higher the concentration of ZnO NP, the more aggregates can be seen. Aggregation occurs because cations of test media bind to ZnO NP where Ag ions are not adsorbed. The behavior of ZnO NP are affected by ion combination and ionic strength. That is, ZnO NP tended to promote aggregation formation by divalent cations in the test media rather than monovalent ones [33]. Thus, if this mechanism is true, it is expected that the synergism observed in MIX I and MIX III, as shown in Figure 5(b) and (d) (the dark regions in the gut that were filled with NPs), can be explained by this mechanism together with toxicity caused by an excessive ionic Zn released from the ZnO NPs.

In general, the CA model is more suitable as a reference model for environmental risk assessment [34] because it provides a more conservative estimation of mixture toxicity. However, the results of the present study showed DR dependent deviation from the CA model with lowest sum of squared residuals (SS), indicating that the CA model is not always the best model to predict the mixture toxicity of AgNO_3_ and ZnO NPs. When ZnO NPs are released into an environment where Ag already exists, it can interact to varying degrees depending on the ratio in the mixture, thus its effect on aquatic organisms can vary. Therefore, to facilitate the use of mixture toxicity studies in a prospective risk assessment, there is a need to improve understanding of how NPs will behave and interact with other co-contaminants existing in aquatic environments under environmentally realistic concentrations.

Since we have reported the mixture nanotoxicity at a whole organism-level in this work, the next step will be an in-depth mechanistic study on the cellular toxicity pathways at the molecular level, associated with tissue-specific toxicodynamics. Furthermore, the exact evaluation of uptake levels of Zn and Ag is required for the future research, under the consideration of time-dependent biodistribution profiles inside *D. magna*.

## 4. Conclusions

The present study showed DR dependent deviation in both reference models. The lowest SS was obtained in the CA-DR model, suggesting that CA-DR would be the model of best fit to describe the pattern of mixture toxicity of ionic Ag and ZnO NPs. Specifically, antagonism occurred when Ag ions became dominant in the mixture, while synergism occurred when ZnO NPs became dominant in the mixture, showing a shift between synergism and antagonism that was dependent on the TU ratio of the mixture constituents. Given that the mixture toxicity cannot be predicted by the general conceptual CA model, further studies on the mixture toxicity of NPs and coexisting contaminants in environmentally realistic concentrations are required.

## Supplementary Material

Supplemental Material
