# Supplementary material for: Quantitative prediction of mixture toxicity of AgNO3 and ZnO nanoparticles on Daphnia magna
Source: Sci Technol Adv Mater. 2020 Jun 16;21(1):333–45. doi: 10.1080/14686996.2020.1766343 (PMC7476544; doi:10.1080/14686996.2020.1766343)

Supplementary Information

Quantitative prediction of mixture toxicity of $AgNO_3$ and ZnO nanoparticles on *Daphnia magna*

Min Jeong Baek[1†], Jino Son[2], Jayoung Park[1], Yohan Seol[1,3], Baeckkyoung Sung[1,3], Young Jun Kim[1,3]*

[1] *KIST Europe Forschungsgesellschaft mbH, 66123 Saarbrücken, Germany*

[2] *Ojeong Ecoresilience Institute, Korea University, 02841 Seoul, Republic of Korea*

[3] *Division of Energy & Environment Technology, University of Science & Technology, 34113 Daejeon, Republic of Korea*

[†] *Present address: Korean Entomological Institute, Korea University, 02841 Seoul, Republic of Korea*

*Correspondence: Young Jun Kim, Ph.D.

Email: youngjunkim@kist-europe.de

Figure S1. Hydrodynamic diameter, zeta potential, and released amount of zinc ion in each of the three types of individual (ZnO NPs alone; ZnO I, II, and III) and mixture ($AgNO_3$+ZnO NPs; MIX I, II, and III) suspensions over incubation time (0, 24, and 48 h). [Representation of the data in **Table 1**]

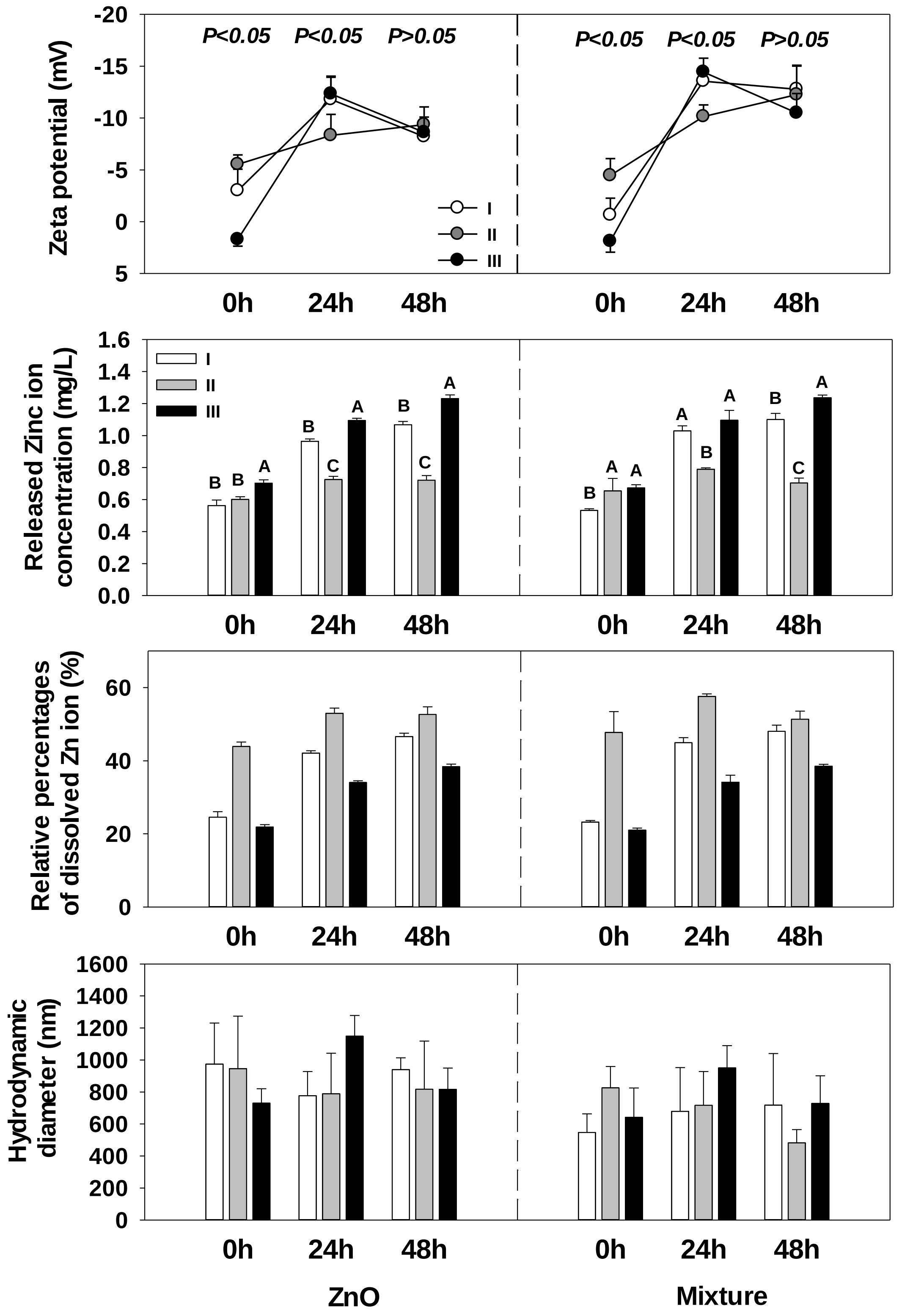

Figure S2. The accumulation of zinc and silver ions in *D. magna*, measured by ICP-MS. The analysis was performed after 6 hours' exposure of *D. magna* to Mixture I, II, III in the same proportion to the neonate experiments, to the highest concentrations of Ag and Zn ("Ag II" and "ZnO III"), and to the control (ISO media). Each error bar represents standard error (n = 3).

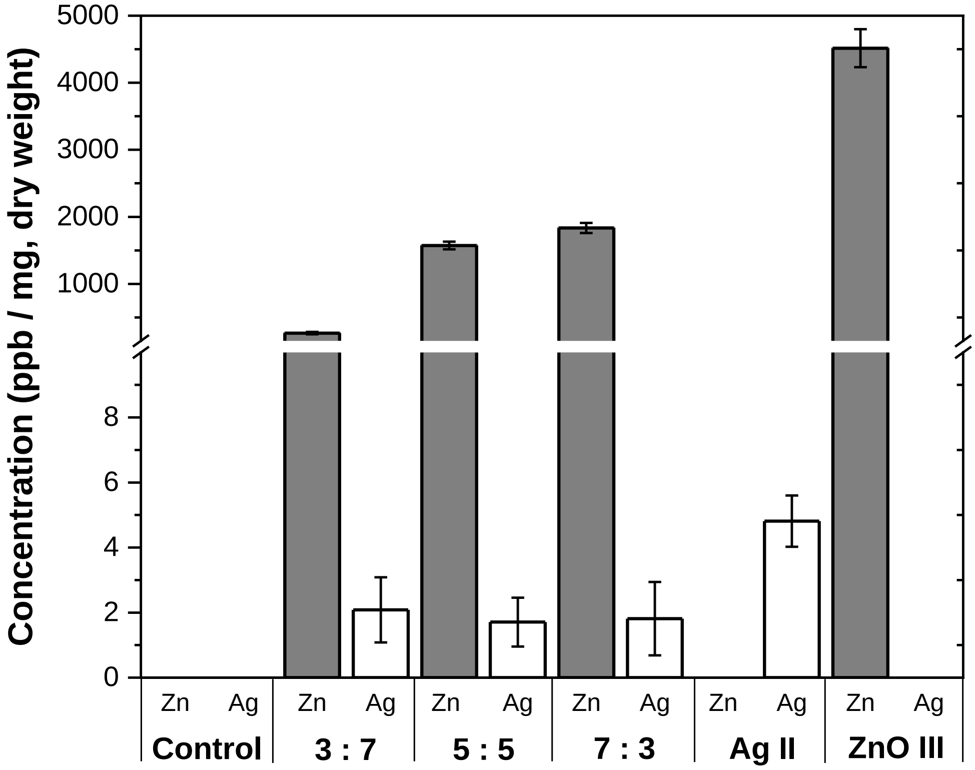

Supplement: Supplemental Material [file TSTA_A_1766343_SM7668.pdf]
